# How effective are programs at managing transition from hospital to home? A case study of the Australian transition care program

**DOI:** 10.1186/1471-2318-12-6

**Published:** 2012-03-14

**Authors:** Leonard C Gray, Nancye M Peel, Maria Crotty, Susan E Kurrle, Lynne C Giles, Ian D Cameron

**Affiliations:** 1 Centre for Research in Geriatric Medicine, The University of Queensland, Level 2, Building 33, Princess Alexandra Hospital, Woolloongabba, Queensland 4102, Australia; 2 Department of Rehabilitation & Aged Care, Flinders University, Repatriation General Hospital, Daw Park, South Australia 5041, Australia; 3 Rehabilitation and Aged Care Service, Hornsby Ku-ring-gai Hospital, Hornsby NSW 2077, Australia; 4 Discipline of Public Health, The University of Adelaide, South Australia 5005, Australia; 5 Rehabilitation Studies Unit, Sydney Medical School, University of Sydney, PO Box 6, Ryde NSW 2112, Australia

## Abstract

**Background:**

An increasing demand for acute care services due in part to rising proportions of older people and increasing rates of chronic diseases has led to new models of post-acute care for older people that offer coordinated discharge, ongoing support and often a focus on functional restoration. Overall, review of the literature suggests there is considerable uncertainty around the effectiveness and resource implications of the various model configurations and delivery approaches. In this paper, we review the current evidence on the efficacy of such programs, using the Australian Transition Care Program as a case study.

**Discussion:**

The Australian Transition Care Program was established at the interface of the acute and aged care sectors with particular emphasis on transitions between acute and community care. The program is intended to enable a significant proportion of care recipients to return home, rather than prematurely enter residential aged care, optimize their functional capacity, and reduce inappropriate extended lengths of hospital stay for older people. Broadly, the model is configured and targeted in accordance with programs reported in the international literature to be effective. Early evaluations suggest good acceptance of the program by hospitals, patients and staff. Ultimately, however, the program's place in the array of post-acute services should be determined by its demonstrated efficacy relative to other services which cater for similar patient groups.

**Summary:**

Currently there is a lack of robust evaluation to provide convincing evidence of efficacy, either from a patient outcome or cost reduction perspective. As the program expands and matures, there will be opportunity to scrutinise the systematic effects, with lessons for both Australian and international policy makers and clinical leaders.

## Background

Internationally, short hospital lengths of stay and a high demand for post-acute care have led to new models of care for older people that offer coordinated discharge, ongoing support and often a focus on functional restoration. Transition Care is a term that encompasses such services and is defined as a set of actions designed to ensure the coordination and continuity of healthcare as patients transfer between different levels of care and among a diverse range of providers, services and settings [1,2].

Reform programs which enhance clinical integration and continuity of care include innovative models for integrated service delivery and care [3,4]; establishment of new "cross-border" roles for care coordinators to improve transitions across healthcare settings [1,5]; and case managers such as community 'matrons'[6]. It is probable that those most likely to gain from these interventions are older people at high risk of admission to hospital or residential aged care, including those with multiple medical problems, functional deficits, cognitive impairment and social and emotional problems [5].

Systematic reviews examining discharge planning [7,8] suggest that coordinated discharge of older people across hospital-community interfaces reduces hospital readmissions and brings about small reductions in hospital length of stay. Evidence on more complex hospital substitution programs is encouraging with systematic reviews on home-based care [9], early discharge programs [10] and best place of care [11] all suggesting that care can be delivered outside hospital and achieve comparable outcomes. However, cost effectiveness of different models of transition care has been the focus of relatively few rigorous studies [12].

While the intent of transition care is to provide continuing quality care in less intensive care settings in order to meet the needs and priorities of older adults with multiple chronic conditions, economic incentives often drive decisions to move older people into certain settings. A common problem noted in the literature in all countries is that payments are often based on site of care rather than type of care. If a clinical intervention delivered in a specialist setting (e.g. rehabilitation unit) has better outcomes than the same intervention being offered in a non specialist setting (e.g. residential aged care) it may be the staff mix and not the location that is the important difference [11]. The inefficiencies associated with differing sites of care may be related less to the sites than to the staffing patterns and different cultures at the various locations. Overall review of the literature suggests there is considerable uncertainty around the effectiveness and resource implications of the various model configurations and delivery approaches.

Improving transitions for older people across care settings to ensure coordination and quality of long term health care is a global problem faced by health policy makers [13], as is addressing healthcare infrastructure and workforce issues that are inadequate to meet the needs of an aging population [14]. In Australia, as in other countries, older people account for a disproportionate share of health care expenditures, based on a comparison with other age groups. Resources servicing the care needs of older Australians include hospitals, which provide acute and subacute services such as rehabilitation and geriatric evaluation and management beds, and aged care services which include residential aged care facilities and community home care [15].

The Australian Transition Care Program was established at the interface of the acute and aged care sectors with particular emphasis on transitions between acute and community care [15]. In this paper, we describe the program and the evidence of its efficacy.

## Discussion

### The Australian transition care program

The Transition Care Program (TCP) was announced in 2004 as part of the Australian Government's "Investing in Australia's Aged Care: More Places, Better Care". TCP targets older Australians at the conclusion of an acute hospital episode. The stated aims are to provide care that is goal-oriented, short term, therapy focused, and necessary to complete the care recipient's restorative process, optimise their functional capacity, and assist the older person and their families to make long term arrangements for care [16]. The average duration of care is 7 weeks, with a maximum duration of 12 weeks, that may in some circumstances be extended by a further 6 weeks.

Transition Care (TC) is legislated under the Aged Care Act 1997 and in the 2004-5 Federal Budget the Australian Government committed to providing 2,000 flexible aged care places for TC, with a proportion allocated to each state and territory broadly in line with their proportion of people aged 70 years and over. In the 2008-09 Budget, the Government committed a further A$3 million over four years for TC, increasing the total number of TC places for older people after a hospital stay from 2,000 to 4,000. In the context of the Australian population aged 70 years and older, TCP offers 1.0 place per 1000 older persons. This complements other services for older people, with approximately 25% of those aged 70 years and older making some use of aged care and most using care provided in their own homes [17]. Recent estimates suggest that at a national level there are approximately 84.2 residential care places, 17.9 community care packages, 25.7 acute care beds, and 3.0 subacute beds per 1000 persons aged 70 or older [15]. Importantly, there is considerable heterogeneity between different regions of Australia in the number of TC and other aged care packages per 1,000 older persons.

TC is jointly funded by the Commonwealth and State/Territory Governments. Implementation is undertaken by State/Territory health departments, in some cases through aged care organisations, against a set of key requirements [18]. A strength of the program is that TC places are flexible care places under the *Aged Care Act 1997 *which can therefore be offered in either a residential aged care facility or community (home) setting, or a combination of both. The majority (over two thirds) of operational TC places are community-based [19], but this varies by state. The staff mix providing TC services varies across Australia and can be provided by state funded health services or by aged care organisations, depending on the preferences of a State Department of Health or the local area.

To be eligible for TC, older people have completed their acute and (inpatient) sub-acute episode of care, are medically stable and ready for discharge; have been assessed by an Aged Care Assessment Team (ACAT) and in the absence of an alternative, would be eligible for either high or low level residential aged care; and would, in their own and their carer's opinion and on the advice of the health professionals in charge of their care, benefit from a period of care in a non-hospital environment to optimise their functional capacity [20]. The program is implemented within a health context where older people across Australia have variable access to rehabilitation and geriatric hospital beds. The level of access influences recipient selection, with TC in some areas accepting patients who might have been considered suitable for inpatient rehabilitation programs had they been available.

The program offers a wide array of services including, but not limited to, case management, nursing care, social work, physiotherapy, occupational therapy and personal care support services such as assistance with bathing. Services are offered for up to 12 weeks after discharge from hospital. The provision of primary medical care to a TC recipient is undertaken by the person's General Practitioner, in consultation with the Geriatrician or Rehabilitation Medicine Physician where possible [20].

### Effectiveness of TCP against stated objectives

In general, studies of the TCP program to date are not robust, making it difficult to draw conclusions on effectiveness and cost-effectiveness of this model of post-acute care.

TCP is intended to:

• enable a significant proportion of care recipients to return home, rather than prematurely enter residential aged care

• optimize their functional capacity

• reduce inappropriate extended lengths of hospital stay for older people[16].

A national evaluation of the TCP [19] addressed these outcome objectives. The findings are summarised in Table 1.

**Table 1:** Evaluation of Transition Care

Objective	Evaluation Findings
To defer admission to residential aged care	Of the initial 2,443 people approved for TC between 1 October 2006 and 31 March 2007, 1,204 (49%) received TC only in a community setting, 1,026 (42%) only in a residential setting, and 213 (9%) in both a community-based and a residential-based setting. By six months after entering the program, 47% had been readmitted to hospital at least once, 28% had been admitted to residential aged care for long-term care and 14% had died. Those TC recipients who received the program in a residential care setting only, were more likely to remain in residential aged care (n = 595 (58%)) or die (n = 209 (20%)) by six months. An audit conducted early in the program's implementation [21] confirmed that the residential-based services were providing packages to older people with severe disability and more complex care needs, who generally remained in residential aged care following completion of their TC episode.
	Other factors associated with increased risk of residential aged care admission post TCP included increasing age (Odds Ratio (OR):1.05 (95% Confidence Interval (CI): 1.02-1.07)) and lower Modified Barthel Index (MBI) on admission (OR: 0.99 (95% CI: 0.98-0.99)) while increased hours of allied health services provided as part of TCP reduced the risk of admission (OR: 0.79 (95% CI: 0.67-0.94)).^a^
	When the outcomes of the people who received TC were compared with other frail groups discharged from hospital in the same time period, the risk of admission to residential aged care in the six months post TCP approval was higher in the two control groups than among TC recipients overall (Control 1 OR: 1.9 (95% CI:1.5-2.3); Control 2 OR: 1.2 (95% CI: 1.0-1.4)).^a^

To optimize functional capacity	Evidence for a functional outcome of TCP is routinely assessed by the Modified Barthel Index (MBI), measured at admission and discharge. The national evaluation showed the average MBI at admission to TC was 64.3 units and at discharge was 76.9 units, representing an improvement of 12.5 units. However, without comparison groups, it is difficult to determine if the TCP program promotes accelerated recovery from newly acquired disability, compared with traditional approaches (including inpatient sub-acute hospital, day-hospital and community rehabilitation programs), and if such recovery is sustained over the medium or longer term [16].

To minimize inappropriate extended hospital lengths of stay	The national evaluation showed that the median length of stay for the index hospitalisation varied considerably between jurisdictions, making differences between TCP and control groups difficult to interpret. An earlier study assessing the effectiveness of moving patients who were waiting in hospital for a residential aged care bed to an off-site transition care facility [22] suggested that when all bed-days were counted, such units had system efficiency problems [15]. Although transferred patients 'saved' a median 11 (95% CI: 6-16) hospital bed-days, it took a median of 21 days longer (95% CI: 6-27) for them to be admitted to a residential aged care facility than those in the usual care group [22].^b^

^a ^Based on multiple logistic regression analyses that adjusted for cognitive status, total number of ADL items for which help was needed, availability of a co-resident carer, and Charlson comorbidity index at initial hospital stay.

^b ^Based on Mann-Whitney U-tests.

Other outcomes of relevance to older people may involve choice, inclusion in decisions, and participation of families in care planning processes [20]. Although comparatively little work has been done on older people's priorities in the configuration of health services, a survey of TC recipients and carers in the national evaluation found that TCP provided additional treatment and care options following hospitalisation that were highly valued by patients and their families. High levels of satisfaction with TC were reported, with 93% of carers satisfied or very satisfied with the services their relative/friend received [19].

### Cost effectiveness of transition care

Some of the key drivers of this program, at a policy level at least, are financially oriented. These include substitution of hospital beds with in-home programs, step down to an interim care setting rather than remaining in a hospital bed, and prevention of (potentially costly) hospital readmissions and admissions to residential aged care for long-term care.

The program is relatively expensive. Currently funded on an "occupied place day" basis, the average per episode cost over the last three years was A$12,400, with a reported length of stay of 55 days giving an average cost per day of A$225 [23]. Of this, the user may be asked to contribute a maximum of A$442.75 for community-based transition care (calculated on a daily rate of 17.5% of the single pension) or A$2,125.75 for residential-based transition care (calculated on a daily rate of 84% of the single pension). It is important to note that the Australian Government only determines the maximum fee that can be charged; however, it does not mandate what amount is charged across the country. Each jurisdiction has differing policies relating to client contributions for transition care, and therefore the amounts charged may vary significantly across Australia.

Our own estimates of potential cost-effectiveness suggest that, even assuming the most optimistic scenario for a TCP recipient (that is early hospital discharge, reduction in risk of readmission, decreased utilisation of community health services and delayed entry into residential care), the maximal cost off-sets would amount to approximately A$6,100. Such maximal cost off-sets are not sufficient to make the TCP cost-saving or even cost-neutral. The expense may be justified if the intervention can be shown to achieve certain goals such as lessening the 'bed blocker' effect and reducing the demand for residential aged care facilities, even if health gains are not consistently realised.

Of course, there are political and sectoral drivers of this program. This is particularly the case in relation to governance of hospitals and residential aged care facilities in Australia. Hospitals, and their governing organisations, have no responsibility for the cost of operations of the long term residential care facilities, and only partial responsibility for the TCP. They, therefore are inclined to perceive financial gain if patients are moved promptly out of hospital, even if the overall system-wide cost is increased.

## Summary

The Australian TCP is an example of a wide-scale effort to improve patient care and systems efficiency at discharge from hospital, for patients with functional impairment and substantial risk of readmission or entry to residential aged care. In particular, there is potential to enhance the rate of patient recovery, to accommodate or rehabilitate new disability, and to avoid hospital readmission and entry to long term care. Broadly, the model is configured and targeted in accordance with programs reported in the international literature to be effective. The early evaluations suggest good acceptance of the program by hospitals, patients and staff. The program clearly admits patients with significant disability, with high risk of readmission to hospital, or risk of imminent entry to residential care. It seems, for the most part, to be well targeted to those patients with high risk of undesirable outcomes, which creates a real opportunity for important patient and system level gains.

There is, however, a lack of robust evaluation to provide convincing evidence of efficacy either from a patient outcome or cost reduction perspective. The reported evaluations were retrospective, and suffered from a lack of well matched historical or contemporary control groups. Given the high patient level cost of the program, robust evidence for efficacy should be demanded. As the program matures, further research could provide valuable insight into how such a systematic evaluation might reshape activity at the acute - aged care interface.

Ultimately, however, the program's place in the array of post-acute services should be determined by its demonstrated efficacy relative to other services which cater for similar patient groups. Therefore, its final place in an integrated health system might look quite different to its current status in many jurisdictions, particularly where there are perverse incentives to use the program, or marked deficiencies in supply of conventional rehabilitation and geriatric programs. As the program expands and matures, there will be opportunity to scrutinise the systematic effects, with lessons for both Australian and international policy makers and clinical leaders.

## Abbreviations

CI: Confidence interval; MBI: Modified barthel index; OR: Odds ratio; TCP: Transition care program; TC: Transition care

## Competing interests

The authors declare that they have no competing interests.

## Authors' contributions

As Chief Investigators on the program grant *Transition Care: Innovation and Evidence*, authors IDC, MC, LCG (Gray), SEK and LCG (Giles) developed the study design concept and contributed to the writing. LCG (Giles) and MC undertook a national evaluation of Transition Care. NMP undertook the literature searches and contributed to drafting the manuscript. All authors read and approved the final manuscript.

## Pre-publication history

The pre-publication history for this paper can be accessed here:

http://www.biomedcentral.com/1471-2318/12/6/prepub
